# Multi-omics dataset of bovine mammary epithelial cells stimulated by ten different essential amino acids

**DOI:** 10.1038/s41597-024-03123-4

**Published:** 2024-03-12

**Authors:** Lianbin Xu, Xiaowen Wang, Xiuli Li, Huawei Liu, Jinshan Zhao, Dengpan Bu

**Affiliations:** 1 College of Animal Science and Technology, Qingdao Agricultural University, Qingdao, 266109 P. R. China; 2 College of Veterinary Medicine, Qingdao Agricultural University, Qingdao, 266109 P. R. China

**Keywords:** Metabolomics, Transcriptomics

## Abstract

Application of high-throughput sequencing and screening help to detect the transcriptional and metabolic discrepancies in organs provided with various levels of nutrients. The influences of individual essential amino acid (EAA) administration on transcriptomic and metabolomic profilings of bovine mammary epithelial cells (BMECs) were systematically investigated. A RNA sequencing and liquid chromatography-tandem mass spectrometry generated a comprehensive comparison of transcriptomics, non-targeted metabolomics and targeted amino acids profilings of BMECs with individual EAA stimulation by turn. The sequencing data and raw LC-MS/MS data of samples were presented in the databases of Gene Expression Omnibus, MetaboLights and Figshare for efficient reuse, including exploring the divergences in metabolisms between different EAAs and screening valuable genes and metabolites regulating casein synthesis.

## Background & Summary

Milk protein from mammary gland of dairy cows is a valuable protein source available for human consumption^1^. However, insufficient utilization of genetic potential restricts dairy’s competitiveness and weakens efforts to decline investments into food production^2^. Although it varies greatly, the efficiency of dietary nitrogen conversion into milk is only about 25% in lactating cows, which results in a huge pecuniary loss and a series of environmental pollutions^3^.

Given that, nitrogen intake has a significant correlation to nitrogen excretion^4^. Dairy cows are likely to be supplied protein that exceed their requirements resulting from unclear definitions of amino acid (AA) needs. The bovine mammary gland is a forceful milk protein synthesizing factory. Present diet formulation is according to the single limiting nutrient theory proposed by von Liebig, which suggested that AA transfer from lumen to milk follows a stationary efficiency until needs are satisfied^5^. However, the observations that several AAs regulated cell signaling pathways^5,6^ and the changeable AA transport activity of mammary gland indicate the mutable efficiency of AA utilization that violates the supposition raised by Mitchell and Block^7^. Therefore, more data about the individual AA metabolism in mammary gland is required to improve the predictions of AAs requirements for dairy cows.

When dairy cows were fed an average diet of 17.8% crude protein (CP), only 25% of dietary nitrogen is recovered in milk^3^, but this efficiency increased to 30% under a grass-based diet with a well-balanced supplement of histidine, lysine, methionine, and leucine to a 15% CP diet^8^. Similarly, Zhao *et al*. reported that lactating cows fed 12% CP diet supplemented with isoleucine, leucine, methionine and threonine had similar milk productions relative to those fed 16% CP diet^9^. These results indicated that a reasonable strategy to elevate postabsorptive nitrogen efficiency is to reduce present percentages of dietary nitrogen and add specific essential AAs (EAA), which requires a better understanding and comparison of different EAA conversions in mammary gland.

Similar to the low-protein diet supplemented with EAAs in dairy cows, single EAA addition to the standard medium devoid of total EAAs had been used to explore the influences of single EAA on milk protein synthesis *in vitro*^1,5,10^. Gao *et al*. found that cultivation with standard medium devoid of all AA and supplemented with histidine, lysine, methionine, and leucine for 6-h had different effects on β-casein synthesis^11^, respectively. Appuhamy *et al*. reported that media lack of total EAA and individually supplemented with arginine, isoleucine, leucine, methionine, or threonine exerted various influences on mTOR signaling pathways that regulate the rates of translation initiation and elongation^5^. These results indicated that different individual EAA addition under low total AA/EAA settings had diverse effects on casein synthesis. Besides, additions of several EAAs, such as arginine, leucine and branched-chain AAs were also used to improve the health status of humans with preeclampsia, sarcopenia and cirrhosis^12–14^, respectively. However, previous studies usually focused on few EAAs, and information about the systematic comparison of mammary metabolisms between all individual EAAs is relatively limited. Therefore, transcriptomic and metabolomic profilings as well as the AA concentrations of bovine mammary epithelial cells (BMECs) came from 72 samples with various single EAA availability (0 or standard concentration in medium for each EAA) were systematically compared in this study based on TruSeq Stranded mRNA LTSample Prep Kit (Illumina) and liquid chromatography-tandem mass spectrometry (LC-MS/MS). All of data were deposited in Gene Expression Omnibus^15,16^, MetaboLights^17–19^ and Figshare^20,21^ repositories.

The public availability of this dataset helps to promote researches in biochemistry of EAA regulating milk protein synthesis, transcriptomic and metabolomic comparisons between different studies or explorations into repetitiveness of data analysis in multi-omics. Given that this study has a horizontal design, this dataset can be used in multiple perspectives. First, these data presented a landscape of transcriptomic and metabolomic profiles in BMECs with different individual AA supplies, which contributes to investigate the discrepancies between individual EAA metabolisms. Second, it helps researchers to study the pathways by which EAAs mediate BMECs function and milk protein synthesis. Third, data can be applied for optimizing mammary mathematical models to predict the responses of milk protein synthesis to EAA administrations and contributing to screening key genes and metabolites with potential breeding values.

## Methods

### Study design

Experimental protocols were ratified by Welfare and Health Committee of Qingdao Agricultural University. Primary BMECs were isolated as described previously^22^. Briefly, mammary gland tissues from healthy dairy cows were cut into 1mm^3^ pieces in D-Hanks solution (Solarbio, Beijing, China) containing 1% penicillin-streptomycin (Sigma-Aldrich, MO, USA). Following washing with PBS buffer three times, the pieces were incubated at 37 °C and 5% CO_2_. The tissues were removed when the cells isolated from tissues reached 80% confluence. BMECs and fibroblasts were divided according to their different sensitivity to 0.15% trypsin plus 0.02% EDTA (Sigma-Aldrich, MO, USA). BMECs (6 passages) were grown in DMEM-F12 medium (Gibco, NY, USA) including 1% L-glutamine and 10% (v/v) fetal bovine serum (Gibco, NY, USA) as well as 5 μg/mL insulin and 1% penicillin-streptomycin (both from Sigma-Aldrich, MO, USA) at 37 °C and 5% CO_2_.

**Fig. 1:**
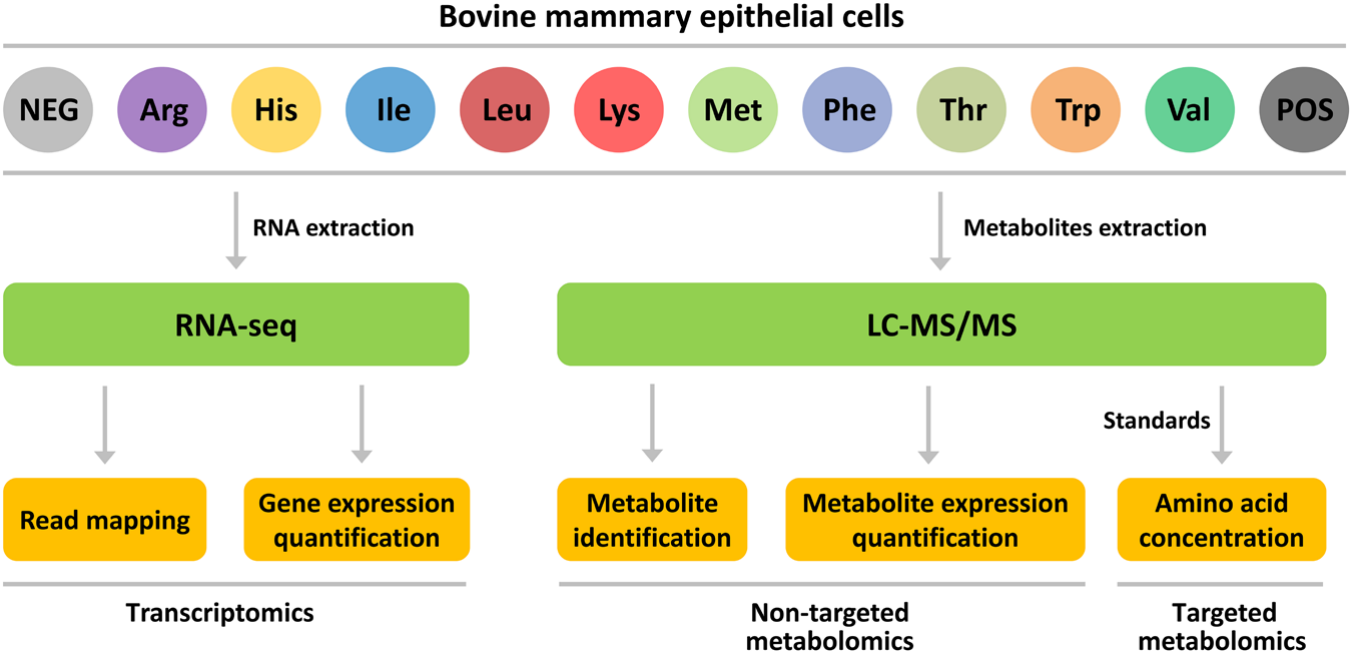
The experimental design and workflow to acquire data of transcriptomics, non-targeted metabolomics and targeted amino acids profilings in BMECs. Complete DMEM-F12 medium used for BMECs was the positive control (POS) treatment, while DMEM-F12 medium without total essential amino acid served as the negative control (NEG) treatment. Ten treatments were NEG individually supplemented with L-arginine, L-histidine, L-isoleucine, L-leucine, L-lysine, L-methionine, L-phenylalanine, L-threonine, L-tryptophan or L-valine, respectively (*n* = 6). Individual EAA was supplemented to achieve a concentration equal to that in POS.

The experimental design was shown in Fig. 1. For exploring the transcriptomic and metabolomic responses to diverse EAA stimuli, 0.5 × 10^7^ BMECs were seeded in a 12-well plate. After nearly 48-h cultivation, the cells were serum-starved overnight when they reached a 90% confluence and had a aggregates-like formation, and then subsequently assigned to 1 of 12 treatment media (n = 6). Complete DMEM-F12 medium (Gibco, NY, USA) containing 0.70 L-arginine, 0.15 L-histidine, 0.42 L-isoleucine, 0.45 L-leucine, 0.5 L-lysine, 0.12 L-methionine, 0.22 L-phenylalanine, 0.45 L-threonine, 0.04 L-tryptophan, and 0.45 L-valine (all in mmol/L) serves as the positive control (POS) treatment, while DMEM-F12 medium without total EAA served as the negative control (NEG) treatment. Ten treatments were NEG individually supplemented with L-arginine, L-histidine, L-isoleucine, L-leucine, L-lysine, L-methionine, L-phenylalanine, L-threonine, L-tryptophan or L-valine (Sigma-Aldrich, MO, USA), respectively. Individual EAA was supplemented to achieve a concentration equal to that in the POS treatment. After 6-h treatment, cell samples were collected simultaneously and kept at −80 °C for subsequent analysis.

### Sample preparation

Total RNA of treated cells were collected with TRIzol reagent (Invitrogen, Carlsbad, CA, USA). The quantity and quality (optical density at 260/280 nm = 1.8–2.0) of RNA were measured using a biophotometer (Eppendorf, Hamburg, Germany) and agarose gel electrophoresis for analyzing 28 S and 18 S rRNA subunits.

For non-targeted and targeted metabolomics, the metabolites of 1 × 10^6^ cells were taken from each treatment using 1 mL of reagent including methanol, acetonitrile and water (2:2:1, v/v/v). The BMECs solutions were then vortexed for 1 min, following by ultrasonicating for 0.5-h at 4 °C and for 1-h at −20 °C to precipitate protein. A vacuum centrifuge was adopted to dry the supernatant of solution after a centrifugation with 14,000 rcf for 20 min, and then maintained at −80 °C until latter use. At last, the precipitation after drying was dissolved in 0.1 mL acetonitrile/water (1:1, v/v) and adequately vortexed, and then followed a 20 min centrifugation with 14,000 rcf to obtain supernatants for subsequent LC-MS/MS analysis.

### Total RNA sequencing

Poly-T oligo-attached magnetic beads were applied to purify mRNA. The synthesis of first-strand cDNA was conducted by random hexamer primer and M-MuLV Reverse Transcriptase(RNase H-), and the second-strand cDNA was generated with DNA Polymerase I and RNase H. Adaptor having hairpin loop structure were ligated after the adenylation of 3’ ends of DNA fragments. AMPure XP system was used to purify PCR products. The quality of library was assessed by the Bioanalyzer 2100 system (Agilent Technologies, CA, USA). The library establishments were then sequenced using an Illumina sequencing platform (HiSeqTM 2500) and 150 bp paired-end reads were producted.

### Chromatography

BMECs samples were dissociated on an UHPLC (Vanquish UHPLC, Thermo) along with a Orbitrap. The mobile phase included A = ammonium acetate (25 mM) and ammonium hydroxide (25 mM) in water as well as B = acetonitrile. The process was 98% acetonitrile for 90 seconds and was decreased to 2% during 10.5 min linearly, and maintained for 2 min following by increasing to 98% in 6 seconds. Continuous analysis of samples was conducted arbitrarily to reduce the influences of instrument detection signal fluctuations. Quality control (QC) samples that were composed of aliquots from total samples were operated 3 times before the queue to monitor the column condition and every 6 inserts after that to evaluate discrepancies.

BMECs samples for targeted metabolomics of AA profiling were separated on an Agilent 1290 Infinity LC UHPLC System (Agilent Technologies, CA, USA) using a HILIC column. The mobile phase of composition A includes water, ammonium formate (25 mM) and formic acid (0.1%), and B was 0.1% formic acid in acetonitrile. The process of elution was: 85% B during 0-1 min; B reduced from 85% to 50% (1–11 min) in a linear manner, B was kept at 40% (11.1–12 min), B elevated from 40% to 75% (12–12.1 min), and finally B was kept at 75% (12.1–19 min). Continuous analysis of samples was conducted arbitrarily to reduce the influences of instrument detection measuring undulations. QC samples were operated 3 times before the queue to monitor the column condition and every 6 inserts after that to evaluate discrepancies. Chromatographic retention time was corrected by the blend of standard AA metabolites.

### Mass spectrometry

ESI positive and negative ion modes were adopted in non-targeted metabolomics. BMECs samples were dissociated through UHPLC and underwent mass spectrometry using a Thermo Scientific Orbitrap Exploris 480 (Thermo Fisher Scientific). The ESI source settings were: source temperature, 600 °C; ion source gas 1 (nitrogen), 60; ion source gas 2 (nitrogen), 60; curtain gas, 30; ion spray voltage floating, ±5,500 V. In MS only acquisition, 80–1,200 Da m/z was got by the system coupled with the resolution of 60,000 and the accumulation time of 100 ms. In auto MS/MS acquisition, the system was adjusted to get over the m/z from 70 to 1,200 Da and the resolution was adjusted as 30,000. The accumulation time was adjusted as 50 ms, and exclude time within 4 s.

For targeted metabolomics of AA profilings, ESI positive ion mode was adopted for detection. Mass spectrometry of sampes adopted a 6500/5500 QTRAP mass spectrometer (AB SCIEX, Framingham, MA, USA). The ESI source setting was: ion source gas 1 (nitrogen): 40; ion source gas 2 (nitrogen): 40; curtain gas: 30; source temperature: 500 °C; ion spray voltage floating ±5,500 V. Ion pair was detected using the mode of multiple reaction monitoring.

### Data analysis

The QC and reads statistics in transcriptomics were measured by Trimmomatic^23^. Subsequent analyses were conducted with high-quality clean data. Hisat2 was used for the mapping of *Bos taurus* clean reads to the corresponding reference genome^24^. StringTie (v1.3.3b) was adopted to assemble the mapped reads of each sample^25^. Each transcript’s expression was quantified using the amount of Fragments Per Kilobase of transcript sequence per Millions base pairs sequenced (FPKM) method^26^, and the read counts as well as FPKM value were computed by cufflinks and htseq-count^27^, respectively. The two expression profilings between each treatment and control group were quantified by DESeq R package using nbinom test^28^. Significant differentially expressed genes (DEG) were discriminated when the unigenes have *P* < 0.05 and |log2(fold-change)| > 1.

The raw data files of non-targeted metabolomics were transformed to the format of mzML by ProteoWizard^29^. XCMS program was applied for peak alignment, retention time correction, and peak area extraction^30^. The following settings for peak picking were applied: centWave m/z = 25 ppm, peakwidth = c (10, 60), prefilter = c (10, 100). Bw = 5, mzwid = 0.025, minfrac = 0.5 were applied for peak grouping. Metabolite identification was conducted by MS/MS spectra using an in-house database. To make the metabolomics data reproducible, the relative standard derivation (RSD) of the peaks in the QC samples larger than 30% were filtered out. After normalized to total peak intensity, the processed data were uploaded into SIMCA-P (version 14.1, Umetrics, Umea, Sweden), where it was subjected to multivariate data analysis, including Pareto-scaled principal component analysis (PCA) and orthogonal partial least-squares discriminant analysis (OPLS-DA). Response permutation testing along with 7-fold cross-validation were used to assess the robustness of model. The variable importance in the projection (VIP) value of each variable in the OPLS-DA model was calculated to indicate its contribution to the classification. Significance was measured with an unpaired Student’s t test. Compounds with VIP value > 1 and *P* < 0.05 were considered as differentially expressed metabolites (DEM).

For targeted metabolomics of AA profiling in BMECs samples, multiQuant software was adopted to extract the chromatographic peak area and retention time. AA standards were used for retention time correction and metabolites identification^31^. Compounds having coefficient of variation under 30% cross samples were identified as reproducible measurements. For DEM identification, statistical analyses between two groups for each EAA administration (NEG vs. individual EAA treatment or POS vs. individual EAA treatment) were conducted with fold changes and *P-*values that were obtained according to a Student’s *t* test. Metabolites having *P-*values < 0.05 and VIP > 1 were considered as DEM.

## Data Records

The sequencing data of BMECs with different EAA administrations were presented in Gene Expression Omnibus (https://www.ncbi.nlm.nih.gov/geo) with number of GSE232591^16^. Correlation coefficient matrix among the samples used for transcriptomics was uploaded to Figshare^20^. Raw LC-MS/MS data files for non-targeted and targeted metabolomics in BMECs were uploaded to the MetaboLights^17^ database (http://www.ebi.ac.uk/metabolights) under MTBLS7789^18^ and MTBLS3956^19^, respectively. Metabolic annotation and DEM analysis were present in Figshare^21^.

## Technical Validation

Quality of the sequencing data was evaluated according to the sequence quality, GC content, presence of adaptors and overrepresented k-mers with FastQC. Samples subjected to routine data cleaning to guarantee that no base was called with a Phred quality below 20. Statistical robustness was supported by the 6 biological replicates of each treatment. A number of raw reads came from 12 treatments, ranging from 40,612,706 to 53,705,974 (Table 1). A total of 40,253,216 to 52,418,068 clean reads were kept after trimming and the overall mapping efficiency ranged from 95.92 to 97.71%. These results suggested that the sequencing data has a high quality for subsequent analysis. Correlation of gene expressions between samples is an important parameter to check the experimental reliability. Our data deposited at Figshare^20^ showed that the pearson correlation coefficient (*r*) with a square value between samples were all greater than 0.85, which was a prerequisite for subsequent differential expression analysis.

**Table 1:** Statistics analysis of clean reads mapping onto reference genome.

Sample	Raw reads	Clean reads	Mapping efficiency	Phred> 20 (%)	Sample	Raw reads	Clean reads	Mapping efficiency	Phred >20 (%)
NEG_1	48,526,890	47,549,694	92.61%	96.85	Met_1	46,384,336	45,506,434	93.78%	96.19
NEG_2	43,176,084	42,530,258	92.74%	96.88	Met_2	41,498,318	40,413,838	93.45%	97.31
NEG_3	45,940,308	44,989,364	92.71%	96.73	Met_3	40,793,346	40,266,998	93.33%	97.35
NEG_4	45,406,952	44,616,416	92.71%	96.66	Met_4	43,268,450	427,36,254	93.03%	97.33
NEG_5	50,541,714	49,594,468	92.88%	96.79	Met_5	47,697,704	47,125,238	93.38%	97.35
NEG_6	46,829,238	46,111,516	92.94%	97.00	Met_6	45,551,840	44,703,604	93.52%	96.90
Arg_1	46,390,570	45,987,698	92.96%	97.35	Phe_1	47,526,272	46,582,198	93.02%	96.84
Arg_2	46,964,992	46,211,062	92.11%	97.22	Phe_2	47,428,866	46,399,916	93.30%	96.81
Arg_3	46,015,946	45,668,658	93.15%	96.84	Phe_3	53,705,974	52,418,068	92.90%	96.87
Arg_4	44,911,936	44,367,842	83.80%	97.30	Phe_4	46,669,402	45,601,374	92.69%	96.50
Arg_5	44,816,418	44,285,818	93.62%	97.21	Phe_5	50,288,164	49,017,216	92.47%	96.61
Arg_6	46,753,196	46,193,354	93.47%	97.12	Phe_6	51,493,910	49,931,934	92.55%	96.77
His_1	47,151,788	46,601,100	92.49%	97.10	Thr_1	46,907,428	45,578,642	93.25%	96.67
His_2	45,263,000	44,714,050	92.90%	96.90	Thr_2	53,469,642	52,135,856	93.44%	96.78
His_3	40,612,706	40,253,216	92.46%	97.10	Thr_3	45,859,072	44,557,912	92.85%	96.94
His_4	41,962,688	41,542,730	92.18%	97.25	Thr_4	50,051,988	48,869,674	93.29%	96.82
His_5	47,219,594	46,860,716	92.97%	97.41	Thr_5	47,181,948	46,294,180	93.25%	96.79
His_6	44,961,658	44,630,518	92.75%	97.09	Thr_6	45,299,044	44,021,930	92.32%	96.67
Ile_1	44,409,430	44,143,866	92.82%	97.28	Trp_1	45,961,922	44,819,548	93.18%	96.79
Ile_2	42,285,282	42,018,938	92.79%	96.82	Trp_2	45,661,406	43,839,576	92.11%	97.01
Ile_3	45,644,514	45,232,916	93.15%	96.90	Trp_3	45,596,684	44,770,264	93.58%	97.03
Ile_4	47,122,466	46,714,988	92.97%	96.97	Trp_4	46,588,266	45,742,108	92.20%	97.23
Ile_5	43,589,468	43,218,066	93.24%	97.23	Trp_5	45,209,728	43,980,576	92.38%	97.07
Ile_6	44,043,016	43,591,956	92.39%	96.9	Trp_6	45,515,404	44,238,466	91.39%	97.24
Leu_1	45,489,370	45,012,398	83.91%	97.22	Val_1	46,208,488	44,557,688	92.17%	97.32
Leu_2	46,056,674	45,504,358	93.09%	97.17	Val_2	45,995,318	44,540,748	91.72%	97.19
Leu_3	44,373,600	44,016,548	92.87%	97.28	Val_3	52,639,776	51,529,692	93.29%	95.92
Leu_4	44,005,676	43,663,748	92.79%	97.22	Val_4	44,879,374	43,045,218	93.40%	97.47
Leu_5	42,430,082	42,029,276	92.66%	97.33	Val_5	45,300,600	44,302,592	92.99%	97.10
Leu_6	44,428,110	43,925,098	93.46%	97.47	Val_6	47,799,064	46,034,646	93.95%	97.40
Lys_1	44,060,770	43,658,652	93.07%	97.18	POS_1	47,010,526	46,347,678	93.84%	97.71
Lys_2	45,720,748	45,363,288	93.50%	96.93	POS_2	43,969,636	42,918,656	92.93%	96.97
Lys_3	42,002,798	41,668,272	93.41%	96.57	POS_3	47,616,334	46,756,190	92.58%	97.15
Lys_4	41,221,456	40,887,146	93.18%	96.61	POS_4	47,351,276	45,510,318	91.82%	97.39
Lys_5	47,273,766	46,400,114	93.43%	97.04	POS_5	46,272,932	45,139,976	91.67%	97.25
Lys_6	46,279,044	45,825,968	93.05%	96.95	POS_6	47,241,486	46,172,198	91.83%	97.15

Complete DMEM-F12 medium used for BMECs was the positive control (POS) treatment, while DMEM-F12 medium without total essential amino acid served as the negative control (NEG) treatment. Ten treatments were NEG individually supplemented with L-arginine, L-histidine, L-isoleucine, L-leucine, L-lysine, L-methionine, L-phenylalanine, L-threonine, L-tryptophan or L-valine, respectively (*n* = 6). Individual EAA was supplemented to achieve a concentration equal to that in POS. After 6-h treatment, total RNA was extracted for latter RNA-seq.

**Fig. 2:**
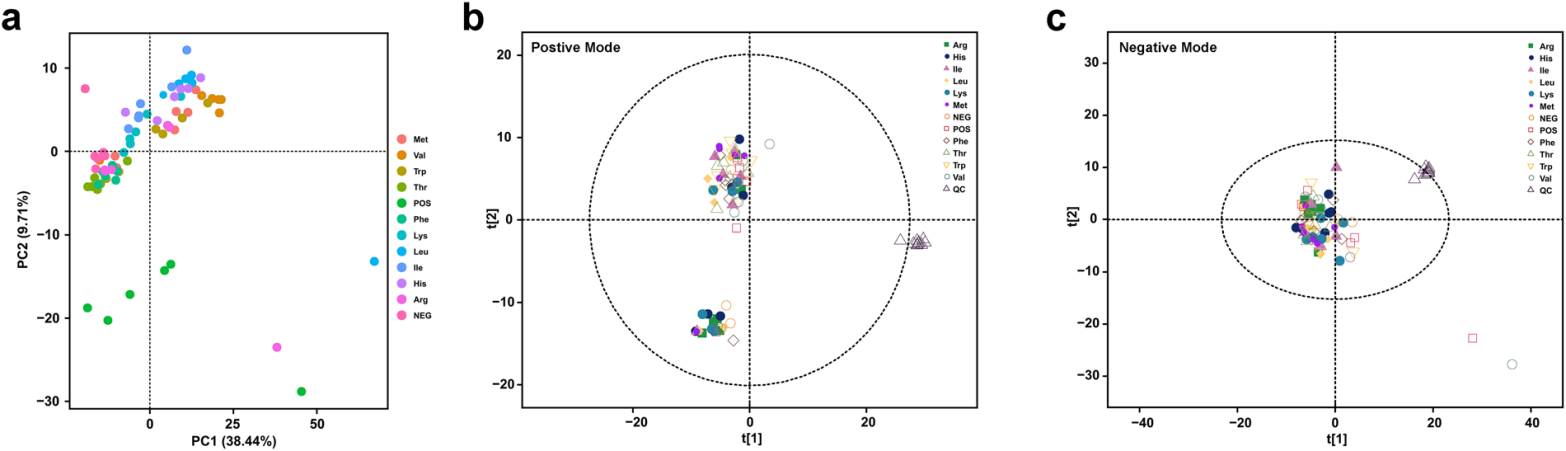
Principal component analysis of the datasets obtained from transcriptomics (**a**) and metabolomics (**b** and **c**). Complete DMEM-F12 medium used for BMECs was the positive control (POS) treatment, while DMEM-F12 medium without total essential amino acid served as the negative control (NEG) treatment. Ten treatments were NEG individually supplemented with L-arginine, L-histidine, L-isoleucine, L-leucine, L-lysine, L-methionine, L-phenylalanine, L-threonine, L-tryptophan or L-valine, respectively (*n* = 6). Individual EAA was supplemented to achieve a concentration equal to that in POS. Quality control (QC) samples in metabolomics that were composed of aliquots from all samples were operated 3 times before the queue to monitor the column condition and every 6 inserts after that to evaluate discrepancies. The distinctive cluster of QC samples indicated that there was no significant variation induced by non-biology in this experiment.

Various methods were used to improve the data quality in mass spectrometry experiments. First, sample extraction, LC-MS derivatization, and MS run followed the randomization sequences. Second, QCs that were made up of different samples were inserted during the measurement process to assess the reliability of data and system stability. Stability of measurement system was also examined using PCA analysis. PCA is a method that gives a overview of the data regarding questions about high variance as well as sample clusters and outliers^32^. QC samples cluster distinctively in Fig. 2, indicating that there was no significant variation induced by non-biology in this experiment.

## Data Availability

FastQC (version 0.11.3, https://www.bioinformatics.babraham.ac.uk/projects/fastqc/) was adopted to check the quality of raw FASTQ sequencing files. Metabolite profiling was analysed with ProteoWizard package (http://proteowizard.sourceforge.net), XCMS Online software (https://xcmsonline.scripps.edu/), SIMCA 13.0 (Umetrics AB, Umea, Sweden) software, MultiQuant software (https://sciex.com/products/software/multiquant-software) and MetaboAnalyst plotform (https://www.metaboanalyst.ca), respectively.
